# Hip disarticulation and external hemipelvectomy for infectious indications: a systematic review

**DOI:** 10.1007/s00590-026-04827-6

**Published:** 2026-06-15

**Authors:** Danil Chernov, Nicholas Frappa, Matthew G. Alben, Thomas Listopadzki, Morgan Dillon, Alexander Kovacs, Ryan Riley, Evgeny Dyskin

**Affiliations:** 1 Jacobs School of Medicine and Biomedical Sciences, Buffalo, USA; 2 Department of Orthopaedics and Sports Medicine, University at Buffalo, State University of New York, Buffalo, USA

**Keywords:** Hip disarticulation, External hemipelvectomy, Lower extremity amputation, Periprosthetic joint infection, Necrotizing fasciitis, Osteomyelitis, Gas gangrene, Infection, Outcomes

## Abstract

**Background:**

Hip disarticulation and external hemipelvectomy are radical lower-extremity amputations typically reserved for oncologic, traumatic, or severe infectious indications. Outcome estimates for refractory infectious cases remain sparse, and prior reports mix case reports, small series, and administrative databases with inconsistent mortality windows and limited functional follow-up.

**Methods:**

This systematic review was reported in accordance with PRISMA 2020 and prospectively registered in PROSPERO (CRD420261277037). PubMed, Embase, Web of Science, and Cochrane CENTRAL were searched from inception through January 3, 2026. Case reports, case series, and retrospective cohort/database studies were eligible when infectious-indication HD or EH cases were separable. Findings were synthesized narratively. Mortality was summarized primarily at the patient level, with sensitivity analyses separating individual case reports, aggregate studies, and the largest registry cohort. Critical appraisal used JBI checklists for case reports/case series and a modified Newcastle-Ottawa Scale for retrospective cohort/database studies.

**Results:**

Forty-nine studies encompassing 289 infectious-indication patients met inclusion criteria. Periprosthetic joint infection was the most common indication (157/289, 54.3%), followed by necrotizing fasciitis (26/289, 9.0%). One national database study contributed 148 patients, and two patients underwent revision from HD to EH, yielding 291 procedures. Thirty-seven early or in-hospital deaths were explicitly reported; because the largest database study suppressed death counts below 11, the patient-level upper-bound mortality estimate was 37 to < 48 deaths among 289 patients (12.8% to < 16.6%), not a precise pooled rate. Excluding the large registry cohort, early mortality was 37/141 (26.2%). Functional outcome was documented for only 67 survivors; 42/67 (62.7%) remained wheelchair-dependent or bedbound, and 25/67 (37.3%) achieved some degree of ambulation.

**Conclusion:**

Hip disarticulation and external hemipelvectomy performed for infection are associated with substantial early mortality and major functional impairment among survivors. Available evidence does not support a single precise pooled mortality estimate because reporting windows, cohort structures, and follow-up duration vary substantially. Functional results are especially uncertain because they are reported in a minority of survivors.

**Supplementary Information:**

The online version contains supplementary material available at 10.1007/s00590-026-04827-6.

## Introduction

Hip disarticulation (HD) and external hemipelvectomy (EH) represent the most radical forms of lower extremity amputation employed in modern surgical practice. While HD entails complete removal of the lower limb at the coxofemoral joint, EH extends resection to include part or all of the hemipelvis [1]. Although historically utilized primarily for oncologic indications [2], these procedures remain critical, life-saving salvage operations for patients with severe, uncontrolled infections. Infectious pathologies necessitating such radical intervention include refractory periprosthetic joint infection (PJI) [3–5], necrotizing fasciitis (NF) [6, 7], chronic osteomyelitis [8, 9], gas gangrene [10, 11], and rare polymicrobial or atypical infections.

Despite their role as life-saving measures, prognosis after HD or EH remains difficult to define. Reported mortality varies widely, from modern series below 7% [12] to historical infection-associated mortality exceeding 50% [10, 13]. This variability likely reflects changes in perioperative critical care, antimicrobial therapy, surgical staging, and source-control timing, as well as differences in whether studies report in-hospital, 30-day, or longer-term mortality. Outcomes following EH for non-oncologic indications are even less clearly defined because available data are often embedded within mixed-indication cohorts dominated by sarcoma and other oncologic resections [14, 15].

Current evidence regarding HD and EH for infection is fragmented, consisting largely of isolated case reports and small case series with heterogeneous reporting of patient characteristics, microbiology, complications, and follow-up. A prior scoping review of HD and hemipelvectomy prostheses highlighted the limited functional and prosthetic literature at these amputation levels [16], but infection-specific survival, microbiology, and mobility outcomes remain incompletely synthesized. Therefore, the purpose of this study was to analyze patient demographics, infectious etiologies leading to surgical intervention, short-term mortality, microbiological findings, and functional status following HD or EH performed as salvage procedures for infection.

## Methods

### Protocol and registration

This systematic review was reported in accordance with the Preferred Reporting Items for Systematic Reviews and Meta-Analyses (PRISMA) 2020 statement [17]. The review protocol was prospectively registered in PROSPERO (ID: CRD420261277037). PRISMA was used as a reporting framework; review methods and eligibility criteria were specified a priori in the protocol.

### Search strategy

A comprehensive systematic literature search was conducted across four databases: PubMed (MEDLINE), Embase, Web of Science, and the Cochrane Central Register of Controlled Trials (CENTRAL). The search encompassed all literature available from database inception to January 3, 2026. The search string utilized combinations of controlled vocabulary and free-text terms, including (“hip disarticulation” OR “external hemipelvectomy” OR “hindquarter amputation”) AND (“infection” OR “sepsis” OR “osteomyelitis” OR “necrotizing fasciitis” OR “periprosthetic joint infection” OR “gas gangrene”). The full reproducible search strategy for each database is provided in Supplementary Appendix 1. Searches were restricted to English-language publications, introducing potential language bias. Reference lists of included full-text articles were manually screened. Protocol deviations were documented: Scopus was not searched in the final strategy, formal forward-citation screening was not performed, and complications and health-related quality of life were not synthesized as prespecified outcomes because reporting was too sparse and heterogeneous.

### Study eligibility

Studies were eligible if they met the pre-specified population, intervention, and study-design criteria. Outcomes were not used as a stand-alone eligibility requirement; instead, each outcome synthesis used the available denominator for that outcome, consistent with systematic-review guidance on eligibility criteria [18].

- *Population*: Patients of any age or sex presenting with a primary severe lower extremity or pelvic infection, including PJI, NF/NSTI, osteomyelitis, gas gangrene, pressure-ulcer-associated infection, or atypical infectious etiologies.
- *Intervention*: Hip disarticulation (HD) or external hemipelvectomy (EH, including hindquarter amputation terminology) performed primarily to achieve source control of infection.
- *Study design*: Case reports, case series, prospective observational studies, retrospective cohort studies, and administrative database studies were eligible. No prospective studies meeting criteria were identified.

Studies were excluded if they were not published in English, did not involve HD or EH, focused on oncologic, traumatic, or ischemic indications without separable infectious cases, represented duplicate/overlapping reports without unique extractable patients, or lacked sufficient postoperative clinical information to determine whether the patient survived the index episode.

### Study selection

All records identified through the database searches were imported into Covidence (Veritas Health Innovation, Melbourne, Australia) for deduplication and screening. Two reviewers independently screened all titles and abstracts. Subsequently, the same two reviewers independently evaluated the full texts of potentially eligible articles against the inclusion criteria. Discrepancies regarding study inclusion or exclusion were resolved through consensus discussion, with adjudication by a third senior reviewer when necessary. Full-text exclusion categories were recorded to improve PRISMA 2020 transparency.

### Data extraction

Data extraction was performed in duplicate by two independent reviewers using a standardized, pre-piloted spreadsheet. For mixed-indication studies, only patient-level data corresponding to procedures performed primarily for infectious causes were extracted when separable. Extracted variables included study design, patient demographics, comorbidities, primary infectious indication, microbiological isolates, surgical intervention (HD vs. EH), staged or revision procedures, antimicrobial regimens when reported, mortality timing, follow-up duration, and functional/ambulatory status. Missing data were not imputed.

### Outcomes, definitions, and data analysis

All analyses were descriptive in nature. The primary mortality endpoint was early or in-hospital mortality, defined as death during the index hospitalization or within 30 postoperative days when timing was explicitly reported. Deaths reported beyond 30 days were tabulated separately and were not combined with the primary early-mortality estimate. Because several aggregate studies reported only study-defined in-hospital mortality, this endpoint should not be interpreted as standardized 30-day mortality. Functional status was categorized from author descriptions as bedbound, wheelchair-dependent, ambulatory with aids, or independent community mobility. Prosthesis use was extracted separately because it may overlap with mobility category. Microbiology was summarized as non-mutually exclusive organism-class reporting; cohort-wide microbiology percentages were not calculated when aggregate studies reported isolate counts rather than patient-level pathogen profiles. Patient-level and procedure-level denominators were kept separate: the analytic cohort denominator was 289 patients, whereas procedure-specific summaries used 291 procedures because two patients required revision from HD to EH. Case reports, aggregate studies, and the largest national database cohort were summarized separately, and sensitivity analyses were performed to evaluate the influence of large registry data and variable mortality windows. No formal meta-analysis was performed because of substantial clinical and methodological heterogeneity. All data management and statistical analyses were performed using R version 4.5.0 (R Foundation for Statistical Computing, Vienna, Austria).

### Risk of bias assessment

Methodological quality and reporting completeness were independently appraised by two reviewers. JBI Critical Appraisal Checklists were used for case reports and case series [19]. Retrospective cohort and administrative database studies were additionally evaluated using a modified Newcastle-Ottawa Scale (NOS) framework [20]. These tools were used to inform interpretation rather than to generate causal effect estimates. Disagreements were resolved by a third reviewer. Because included studies were uncontrolled, retrospective, and heterogeneous, formal GRADE certainty ratings were not calculated; certainty was described narratively across outcomes.

## Results

### Study selection

**Fig. 1:**
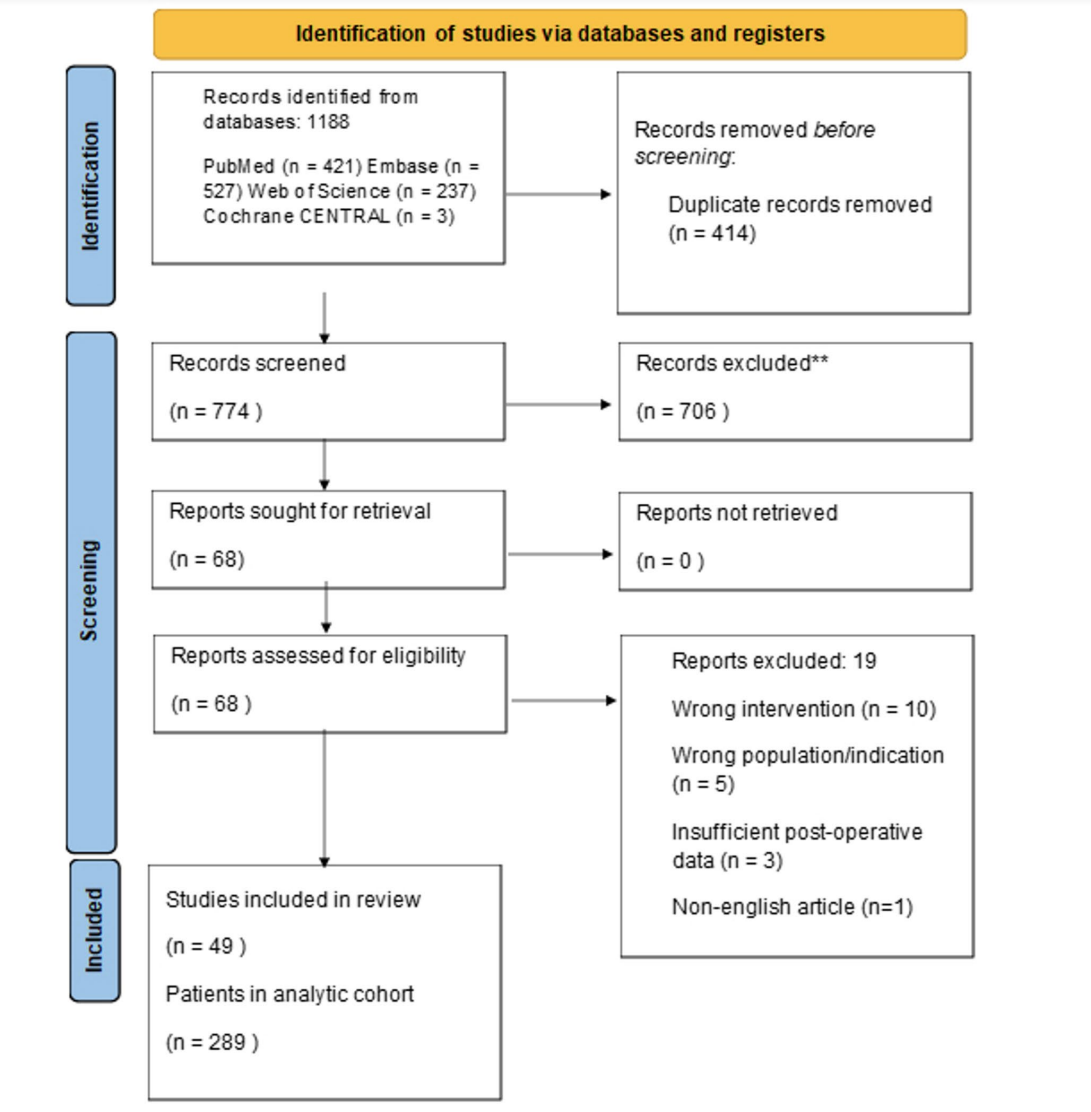
PRISMA flow diagram

The initial literature search identified 1188 database records. Following removal of 414 duplicates, 774 titles and abstracts were screened. Of these, 706 were excluded, leaving 68 reports sought and retrieved for full-text eligibility assessment. Nineteen full-text articles were excluded: wrong intervention (*n* = 10), wrong population/indication or nonseparable infectious data (*n* = 5), insufficient postoperative outcome data (*n* = 3), and non-English publication (*n* = 1). Forty-nine studies were included in the final qualitative synthesis (PRISMA 2020 flow diagram, Fig. 1).

### Study characteristics and patient demographics

The 49 included studies, published between 1977 and 2025, comprised 36 individual case reports (38 patients; Table 1) and 13 aggregate studies (251 patients; Table 2), yielding a 289-patient analytic cohort (Table 3). Sex was extractable at the analytic-cohort level for 260 patients; 131 were male (50.4%) and 129 were female (49.6%). Sex percentages reported only for parent cohorts in Moura et al. [21] and Unruh et al. [13] were not included in the analytic demographic summary. Severe comorbidities were prevalent: diabetes mellitus was present in 51 patients (17.6%), cardiovascular disease in 31 (10.7%), and immunosuppression from chemotherapy, steroids, or systemic disease in 29 (10.0%). Pre-existing pressure ulcers and intravenous drug use were documented in 9.0% and 3.5% of the cohort, respectively.

**Table 1:** Case reports

Authors/Year	Age/Sex	Indications	Microbiology	Anti-microbial regimen	HD^2^ or EH^3^	Experienced mortality	Follow-up duration
Al-Ajlouni et al. [22] / 2014	53/F	Gas gangrene	NR^4^	NR	HD	Yes	Expired 13 days post-op
Alshammary et al. [23] / 2025	26/M	Necrotizing myofasciitis	Rhizopus, Group A Streptococcus	Zosyn, Liposomal Amphotericin B, Tigecycline, Ampicillin, Sulfamethoxazole	HD	No	21 days
Aoki et al. [4] / 2023	24/M	PJI^5^	NR	NR	HD	No	2 years
Balbierz et al. [24] / 2004	45–49^1^/F	NF^6^	Group A Streptococcus	Ampicillin, Gentamicin, Clindamycin, Zosyn	HD	No	Several weeks
Barton et al. [25] / 2024	10/F	Osteomyelitis	NR	NR	HD	No	180 days
Bottner et al. [26] / 2005	70/F* 76/F**	PJI* PJI**	NR* NR**	Gentamicin-loaded spacer, systemic IV antibiotics	HD* HD**	No* No**	19 months* NR**
De Leon et al. [5] / 2020	46/M	PJI	Clostridium septicum	Vancomycin, Zosyn, Cefepime, Flagyl, Linezolid, Cefpodoxime	HD	No	18 months
DiGioia et al. [27] / 1977	58/M	Crepitant cellulitis	Klebsiella pneumoniae	Ampicillin, Gentamicin, IV Cefazolin	HD	Yes	Expired 14 days post-op
Dungan et al. [28] / 2018	62/M	Necrotizing myofasciitis	Clostridium septicum	NR	HD	No	44 days
Hicks et al. [29] / 2022	35/M	NF	Arcanobacterium haemolyticum	IV Flucloxacillin, Zosyn, Clindamycin, Co-amoxiclav	HD	No	21 days
Hofmeister et al. [30] / 2001	69/M	NF	Group A Streptococcus	Penicillin, Ceftazidime, Cefazolin	HD	Yes	Expired 10 days post-op
Hooper et al. [31] / 1977	34/M	Hydatid disease	Echinococcus granulosus, Staphylococcus Aureus	Gentamicin	HD	No	18 months
Horn et al. [32] / 2017	64/F	PJI/ Osteomyelitis	Enterococcus faecalis	IV Ampicillin	HD	No	6 weeks
Huang et al. [33] / 2023	45/M	NSTI^7^	Staphylococcus Aureus, Streptococcus Group G, Peptostreptococcus anaerobius, Haemophilus parainfluenzae	Ceftriaxone, Flomoxef	HD	No	34 months
Kiel et al. [34] / 2011	26/M	Gas gangrene	Clostridium septicum	Lincomycin, Meropenem, Penicillin, Amoxicillin	HD	No	21 days
Kiser et al. [11] / 2014	9/F	Gas gangrene	Clostridium septicum	Zosyn, Vancomycin, Metronidazole, Clindamycin, Penicillin, Meropenem	HD	No	9 months
Krijnen et al. [35] / 2004	63/F* 77/F**	PJI/Osteomyelitis* PJI/Osteomyelitis**	Pseudomonas aeruginosa, coagulase- negative staphylococci* Enterococcus faecalis**	Gentamicin beads*, systemic antibiotics*, systemic antibiotics**	EH* EH**	No* No**	5 years* 1 year**
Lack et al. [36] / 2011	66/F	Osteomyelitis	MRSA^8^, Serratia marcescans, Pseudomonas aeruginosa, Prevotella spp.	Broad-spectrum antibiotics	EH	Yes	Expired 4 months post-op
Lee et al. [37] / 2023	74/M	PJI	NR	Chronic suppressive antibiotics	HD	No	8 months
Logan et al. [38] / 2019	32/M	Gas gangrene	Clostridium perfringens	NR	HD	No	NR
Meirizal et al. [39] / 2025	45/M	Osteomyelitis	Proteus mirabilis	Broad-spectrum IV antibiotics	EH	No	6 months
Miller et al. [40] / 2019	45/F	NF	NR	NR	HD	No	54 months
Mizutani et al. [41] / 2023	70–79^1^/M	Infected hematoma	Staphylococcus aureus, Enterococcus gallinarum, Enterobacter cloacae, Clostridium perfringens, Clostridium innocuum	Broad-spectrum antibiotics	HD	No	34 days
Mulier et al. [42] / 1993	45/M	Gas gangrene	Clostridium septicum	NR	HD	No	NR
Papanikolas et al. [7] / 2020	70/M	NF	NR	NR	EH	Yes	Expired 12 h post-op
Rajeswari et al. [43] / 2009	20/F	NF	Staphylococcus aureus, Beta-hemolytic Streptococcus Group C, anaerobic Streptococcus spp., Bacteroides spp.	Benzylpenicillin, Gentamicin, Metronidazole	HD	No	50 days
Robbie et al. [44] / 2024	60–69/M1	NF	Escherichia coli, Streptococcus constellatus, Fusobacterium necrophorum, Enterococcus faecium	NR	HD	No	NR
Rupp et al. [45] / 2018	51/M	NF	ESBL^8^- Escherichia coli	NR	EH	No	49 months
Shimizu et al. [46] / 2015	32/M	Morel-Lavallée lesion	Citrobacter, Enterobacter, Enterococcus, Pseudomonas aeruginosa	NR	EH	No	24 months
Silveira et al. [47] / 2025	64/F	NF	Streptococcus anginosus, Streptococcus constellatus, Actinomyces funkei, Peptostreptococcus species, Stenotrophomonas maltophilia, Candida tropicalis	Zosyn, Clindamycin, Vancomycin, Fluconazole	HD	Yes	Expired 1 day post-op
Siwach et al. [48] / 2009	51/F	Hydatid disease	Echinococcus granulosus	Albendazole	EH	Yes	Expired 1 month post-op
Tan et al. [49] / 2021	43/F	Melioidosis	Burkholderia pseudomallei	NR	HD	Yes	Expired 1 day post-op
Tsagozis et al. [50] / 2015	56/M	Hydatid disease	Echinococcus granulosus	Albendazole, Praziquantel	EH	No	12 months
Vancabeke et al. [51] / 1999	59/M	Osteomyelitis	Staphylococcus aureus, Pseudomonas aeruginosa	NR	HD	No	2 months
Yonezu et al. [52] / 2025	82/M	NSTI	Aeromonas hydrophila	NR	HD	Yes	26 days
Yoshikawa et al. [53] / 2019	61/F	NF	NR	NR	HD	No	189 days

^1^Balbierz et al. [24] reported a woman in her late 40s; Mizutani et al. [41] reported a man in his 70s; Robbie et al. [44] reported a man in his 60s. ^2^Hip Disarticulation. ^3^External Hemipelvectomy. ^4^Not Reported. ^5^Prosthetic Joint Infection

^6^Necrotizing fasciitis ^7^Necrotizing Soft Tissue Infection ^8^Extended-Spectrum Beta-Lactamases

**Table 2:** Aggregate studies

Author/Year	Study Type	Study size | No. of patients included^1^	Age [mean ± SD/range] | Sex [no. of patients (%)]	Indications [no. of patients (%)]	Microbiology [no. of patients (%)]	HD^2^ or EH^3^ [no. of patients (%)]	Experienced Mortality [no. of patients (%)]	Reported Follow-up Duration
Colosimo et al. [54] 2020	Case Series	9|8	[40 ± 12 / 20–60] | 6 M (75%) 2 F (25%)	NSTI [8 (100%)]	NR^4^	HD [8 (100%)]	0	Mean: 54 days (Range 17–220 days)
Correa et al. [8] 2008	Case Series	5|5	[29.2 / 21–39] | 5 M (100%)	Osteomyelitis [5 (100%)]	NR	HD [2 (40%)] EH [3 (60%)]	0	Range: 9.6–120 months
Endean et al. [10] 1991	Retrospective Review	53|26	[47.3 / 19–85] | 11 M (79%) 3 F (21%) * [61.9] / 45–79 | 8 M/67% 4 F (33%) **	NSTI [5 (35.7%)] Decubitus ulcer [3 (21.4%] Femoral osteomyelitis [3 (21.4%)] Gas gangrene [3 (21.4%)] * Infected prosthetic vascular graft [4 (33.3%)] Ischemic infection [4 (33.3%)] Gas gangrene [4 (33.3%)] **	Clostridium spp. [3 (21.4%)] * Clostridium spp. [4 (33.3%)] **	HD [14 (100%) * HD [12 (100%) **	[2 (14%)] * [4 (33%)] **	NR
Korambayil et al. [6] 2021	Case Series	5|5	[48.4 / 32–84] | 3 M (60%) 2 F (40%)	NF5 [5 (100%)]	Mixed anaerobes [1 (20%)] Peptostreptococcus, Group B Streptococcus [1 (20%)] Group A Hemolytic Streptococcus [2 (40%)] Group C Hemolytic Streptococcus [1 (20%)]	HD [5 (100%)] EH [2 (40%)] ***	[2 (40%)]	12 months
Moura et al. [21] 2017	Case Series	16|6	[29–87] | M (56%) F (44%) ****	PJI [4 (66.7%)] Ischemic infection [2 (33.3%)]	Staphylococcus aureus [3 (50%)] Pseudomonas aeruginosa [2 (33.3%)] Enterococcus faecium [2 (33.3%)]	HD [6 (100%)]	[2 (33.3%)]	Mean: 36 months
Nuzzo et al. [55] 1982	Case Series	8|1	[68] | F (100%)	Osteomyelitis [1 (100%)]	Pseudomonas aeruginosa, Serratia marcescens, Enterobacter [1 (100%)]	HD [1 (100%)]	0	NR
Schindler et al. [14] 2023	Case Series	15|7	[50 / 21–66] | 2 M (29%) 5 F (71%)	NF [3 (43%)] PJI [2 (29%)] Stump infection [1 (14%)] Pressure ulcer [1 (14%)]	Staphylococcus aureus [3 (43%)] Pseudomonas aeruginosa [2 (29%)] Enterococcus faecium [2 (29%)] Polymicrobial [7, 100%]	HD [6 (86%)] EH [1 (14%)]	[4 (57%)]	Mean: 22 months (Range: 1 day-8 years)
Schwartz et al. [3] 2020	Retrospective Review	148|148	[< 65 (47%) > 65 (53%)] | 60 M (41%) 88 F (59%)	PJI [148 (100%)]	NR	HD [148 (100%)]	[< 11 (< 7.4%)] *****	NR
Simman et al. [9] 2022	Case Series	4|4	[66 / 60–72] | 2 M (50%) 2 F (50%)	NF [1 (25%)] Stump necrosis [2 (50%)] Osteomyelitis [1 (25%)]	NR	HD [4 (100%)]	[1 (25%)]	3 months
Takahira et al. [56] 2002	Case Series	7|2	[27–60] | 2 M (100%)	Gas gangrene [2 (100%)]	Enterococci, Staphylococcus aureus, Group G Streptococcus, Clostridium perfringens, Escherichia coli, Klebsiella pneumoniae, Proteus vulgaris, Citrobacter diversus, Morganella morganii [2 (100%)]	HD [2 (100%)]	[1 (50%)]	NR
Unruh et al. [13] 1990	Retrospective Review	34|23	[20–95] | M/94% F/6%****	Osteomyelitis [10 (43%)] Unspecified infection [13 (57%)]	NR	HD [23 (100%)]	[12 (52%)]	NR
Wagstaff et al. [57] 2019	Case Series	7|1	[48] | F/100%	NF [1, 100%]	NR	HD [1 (100%)]	0	NR
Zalavras et al. [12] 2009	Case Series	15|15	[48 / 18–82] | 10 M (67%) 5 F (33%)	NF [4 (26.7%)] Gas gangrene [3 (20%)] Wound infection s/p debridement for NF [2 (13.3%)] Osteomyelitis [1 (6.7%)] PJI [2 (13.3%)] Infected iliofemoral bypass graft [1 (6.7%)] Infection and stump breakdown s/p AKA^6^ [2 (13.3%)]	Staphylococcus aureus-oxacillin resistant [5 (33.3%)] Staphylococcus aureus-oxacillin sensitive [3 (20%)] Streptococcus alpha-hemolytic [4 (26.7%)] Enterococcus faecalis [3 (20%)] Staphylococcus epidermidis [1 (6.7%)] Pseudomonas aeruginosa [4 (26.7%)] Acinetobacter baumannii [1 (6.7%)] Proteus mirabilis [1 (6.7%)] Other Gram-negative rods [3 (20%)] Microaerophilic streptococcus [5 (33.3%)] Prevotella intermedia [3 (20%)] Peptostreptococcus [1 (6.7%)] Fusobacterium [1 (6.7%)] Propionibacterium acnes [1 (6.7%)] Bacteroides [1 (6.7%)] Clostridium perfringens [1 (6.7%)]	HD [15 (100%)]	[1 (6.7%)]	NR

^1^Only patients with infectious indications for HD^2^ or EH^3^ were included for analysis and table reporting

^2^Hip disarticulation

^3^External hemipelvectomy

^4^Not reported

^5^Necrotizing fasciitis

^6^Status-post above-knee amputation

*Characteristics of infection group in review by Endean et al. [10]

**Characteristics of infection and ischemia group in review by Endean et al. [10]

***Two of three remaining survivors underwent reoperation with EH/hindquarter amputation post-HD in series by Korambayil et al. [6]

****Moura et al. [21] and Unruh et al. [13] reported sex distributions for their full parent cohorts rather than the infectious analytic subsets; these parent-cohort percentages were not used in Table 3

*****Mortality is reported as < 11 due to NIS database restrictions on small cell sizes, representing a rate of < 7.4% in review by Schwartz et al. [3]

**Table 3:** Patient demographics and clinical characteristics

Variable	Overall Cohort
Age, median [range] (years)	48.0 [9–82]
Sex with analytic-cohort data available (*n* = 260)*	
Male	131 (50.4%)
Female	129 (49.6%)
Comorbidities (*n* = 289)	
Diabetes Mellitus	51 (17.6%)
Immunosuppression	29 (10.0%)
Cardiac/Vascular Disease	31 (10.7%)
Pressure Ulcer	26 (9.0%)
IV Drug Use	10 (3.5%)

*Sex was calculated only among patients for whom sex was extractable within the infectious analytic cohort. Parent-cohort sex percentages from Moura et al. [21] and Unruh et al. [13] were excluded from this denominator

### Infectious indications and surgical procedures

Infectious indications and procedure counts are detailed in Table 4. PJI was the most common indication, accounting for 157 of 289 patients (54.3%). Other indications included NF (26/289, 9.0%), chronic osteomyelitis (12/289, 4.2%), gas gangrene (10/289, 3.5%), NSTI (10/289, 3.5%), hydatid disease (3/289, 1.0%), and other rare etiologies (10/289, 3.5%). Sixty-one patients (21.1%) from aggregate studies had mixed or insufficiently stratified infectious indications. A total of 275 HD procedures and 16 EH procedures were reported across 289 patients; the procedure total exceeds the patient total because two patients in Korambayil et al. [6] required revision to EH after index HD for persistent infection.

**Table 4:** Patient-level infectious indications and procedure counts

Indication category	Patients, *n* (%) *n*/d, d = 289	HD procedures, *n*	EH procedures, *n*	Interpretive note
Periprosthetic joint infection (PJI)	157 (54.3%)	155	2	Patient-level indication available
Necrotizing fasciitis (NF)	26 (9.0%)	23	5	Includes two EH revisions after index HD; procedure counts exceed patient counts
Osteomyelitis	12 (4.2%)	5	7	Includes chronic femoral/pelvic osteomyelitis and pressure-ulcer-associated infection
Gas gangrene	10 (3.5%)	10	0	Includes clostridial and nonclostridial gas gangrene
Necrotizing soft tissue infection (NSTI)	10 (3.5%)	10	0	Reported separately when original study used NSTI rather than NF
Hydatid disease	3 (1.0%)	1	2	Parasitic osseous/pelvic disease with secondary infectious relevance
Mixed or unstratified infectious cohorts	61 (21.1%)	61	0	Aggregate studies did not link all patients to a specific infectious subtype
Other/rare infectious indications	10 (3.5%)	10	0	Includes melioidosis, infected hematoma, Morel-Lavallee lesion, crepitant cellulitis, and other rare etiologies
Total	289 (100%)	275	16	Total procedures = 291 because two patients underwent revision EH after HD

### Microbiology and antimicrobial management

Microbiological data were highly heterogeneous and frequently polymicrobial (Table 5). Patient-level microbiology was available for 29 of 38 individual case-report patients. Within this patient-level subset, gram-positive aerobic or facultative bacteria were reported in 16/29 (55.2%), gram-negative bacteria in 13/29 (44.8%), anaerobes in 12/29 (41.4%), fungi or yeast in 2/29 (6.9%), and parasitic disease in 3/29 (10.3%). These categories were non-mutually exclusive. Aggregate studies often reported isolate counts or study-level organism frequencies rather than patient-level pathogen classes, so cohort-wide microbiology percentages were not pooled.

**Table 5:** Microbiology reporting summary

Organism class	Patient-level case reports with microbiology (*n* = 29), n (%)	Procedure distribution in case reports	Representative organisms
Gram-positive aerobic/facultative bacteria	16 (55.2%)	HD 12; EH 4	Staphylococcus aureus, Streptococcus spp., Enterococcus spp., Arcanobacterium haemolyticum
Gram-negative bacteria	13 (44.8%)	HD 8; EH 5	Pseudomonas aeruginosa, Klebsiella pneumoniae, Escherichia coli, Proteus mirabilis, Aeromonas hydrophila, Burkholderia pseudomallei
Anaerobes / clostridial species	12 (41.4%)	HD 11; EH 1	Clostridium septicum, Clostridium perfringens, Bacteroides spp., Peptostreptococcus spp., Fusobacterium necrophorum
Fungi / yeast	2 (6.9%)	HD 2; EH 0	Rhizopus spp., Candida tropicalis
Parasites	3 (10.3%)	HD 1; EH 2	Echinococcus granulosus / hydatid disease

Aggregate studies frequently reported isolate counts or study-level organism frequencies rather than patient-level pathogen classes; cohort-wide microbiology percentages were therefore not calculated

Antimicrobial regimens were explicitly detailed in case reports (Table 1) but rarely documented in aggregate database studies. Empiric therapies for fulminant infections (NF, NSTI, gas gangrene) frequently utilized broad-spectrum coverage such as piperacillin-tazobactam and vancomycin, often combined with clindamycin for antitoxin effects. Culture-directed regimens included high-dose penicillin G for clostridial species, liposomal amphotericin B for fungal co-infections, and albendazole-based therapy for parasitic disease.

### Mortality rates

Mortality estimates are summarized in Table 6. Thirty-seven early or in-hospital deaths were explicitly reported across the analytic cohort. One additional death occurred 4 months postoperatively and was treated as late mortality rather than part of the primary early-mortality endpoint. The largest administrative database study [3] suppressed exact death counts below 11, so the all-study patient-level early-mortality estimate was 37 to < 48 deaths among 289 patients (12.8% to < 16.6%), rather than a precise pooled rate. When the large registry cohort was excluded, early mortality was 37/141 (26.2%). Case reports alone had 8 early deaths among 38 patients (21.1%), while aggregate studies excluding the registry had 29 deaths among 103 patients (28.2%). These sensitivity analyses demonstrate the influence of study design, reporting window, and large database weighting on the apparent mortality estimate.

**Table 6:** Mortality sensitivity analyses by evidence source and follow-up window

Analysis set	Denominator	Deaths counted	Mortality estimate	Interpretation
Primary patient-level early/in-hospital mortality	289 patients	37 to < 48	12.8% to < 16.6%	Includes NIS small-cell suppression in Schwartz et al. [3]; excludes one death reported at 4 months
All reported mortality regardless of follow-up window	289 patients	38 to < 49	13.1% to < 17.0%	Includes the 4-month postoperative death; not a standardized 30-day rate
Excluding Schwartz et al. national database cohort	141 patients	37 early deaths (38 all reported)	26.2% early (27.0% all reported)	Sensitivity analysis showing influence of the largest registry cohort
Individual case reports only	38 patients	8 early deaths (9 all reported)	21.1% early (23.7% all reported)	Highly vulnerable to publication bias and brief follow-up
Aggregate studies excluding Schwartz et al.	103 patients	29 deaths	28.2%	Includes case series and retrospective cohorts with variable mortality definitions
Schwartz et al. NIS PJI database only	148 patients	< 11 in-hospital deaths	< 7.4%	Administrative database with small-cell suppression and limited clinical granularity

### Functional outcomes in survivors

Functional and ambulatory follow-up was sparsely reported in large retrospective cohorts and was available for only 67 survivors (Table 7), representing 23.2% of the total analytic cohort and a minority of survivors. Follow-up ranged from 21 days to 10 years. Among these 67 patients, 42 (62.7%) remained non-ambulatory or dependent at final reported follow-up, including 33 wheelchair-dependent and 9 bedbound patients. Twenty-five patients (37.3%) achieved some degree of ambulation, usually with aids. Prosthesis use was reported in 13/67 (19.4%), but prosthetic fitting and ambulation were not consistently defined across studies.

**Table 7:** Functional outcomes in survivors

Outcome	*n* (%) among 67 survivors with reported functional status
Non-Ambulatory / Dependent	42 (62.7%)
Wheelchair Dependent	33 (49.3%)
Bedbound	9 (13.4%)
Ambulatory	25 (37.3%)
Ambulatory with Aids	16 (23.9%)
Independent Community Mobility	9 (13.4%)
Prosthesis Use	13 (19.4%*)
Functional mobility status was explicitly reported for only 67 survivors (23.2% of the analytic cohort), with follow-up ranging from 21 days to 10 years. Prosthetic fitting/use was extracted separately and may overlap with ambulatory status.	

### Risk of bias and certainty of evidence

Critical appraisal results are shown in Tables 8, 9 and 10. Case reports generally described patient presentation, intervention, and short-term outcomes, but follow-up duration was often brief or absent. Case series commonly met core JBI reporting criteria but had incomplete long-term follow-up, heterogeneous outcome definitions, and no adjustment for confounding. Modified NOS assessment of retrospective cohort/database studies highlighted additional limitations, including administrative coding dependence, small-cell suppression, lack of patient-level microbiology or functional data, and limited control for illness severity. Overall confidence in mortality and functional outcome estimates remains very low, and results should be interpreted as descriptive rather than inferential.

**Table 8:** Quality assessment of the included case reports using the Joanna Briggs Institute (JBI) critical appraisal checklist for case reports

Reference (author, year)	A1	A2	A3	A4	A5	A6	A7	A8	Total Yes (0–8)
Al-Ajlouni et al. 2014 [22]	Yes	Yes	Yes	No	Yes	Yes	Yes	Yes	***7***
Alshammary et al. 2025 [23]	Yes	Yes	Yes	Yes	Yes	Yes	Yes	Yes	***8***
Aoki et al. 2023 [4]	Yes	Yes	Yes	Yes	Yes	Yes	Yes	Yes	***8***
Balbierz et al. 2004 [24]	Yes	Yes	Yes	Yes	Yes	Yes	Yes	Yes	***8***
Barton, 2024 [25]	Yes	Yes	Yes	Yes	Yes	Yes	Yes	Yes	***8***
Bottner et al. 2005 [26]	Yes	Yes	Yes	Yes	Yes	Yes	No	Yes	***7***
De Leon et al. 2020 [5]	Yes	Yes	Yes	Yes	Yes	Yes	Yes	Yes	***8***
DiGioia et al. 1977 [27]	Yes	Yes	Yes	Yes	Yes	Yes	Yes	Yes	***8***
Dungan et al. 2018 [28]	Yes	Yes	Yes	Yes	Yes	Unclear	Unclear	Yes	***6***
Hicks et al. 2022 [29]	Yes	Yes	Yes	Yes	Yes	Yes	No	Yes	***7***
Hofmeister et al. 2001 [30]	Yes	Yes	Yes	Yes	Yes	Yes	Yes	Yes	***8***
Hooper et al. 1977 [31]	Yes	Yes	Yes	Yes	Yes	No	Yes	Yes	***7***
Horn et al. 2017 [32]	Yes	Yes	Yes	Yes	Yes	Yes	No	Yes	***7***
Huang et al. 2023 [33]	Yes	Yes	Yes	Yes	Yes	Yes	Yes	Yes	***8***
Kiel et al. 2011 [34]	Yes	Yes	Yes	Yes	Yes	Yes	Yes	Yes	***8***
Kiser et al. 2014 [11]	Yes	Yes	Yes	Yes	Yes	Yes	Yes	Yes	***8***
Krijnen et al. 2004 [35]	Yes	Yes	Yes	Yes	Yes	Yes	Yes	Yes	***8***
Lack et al. 2010 [36]	Yes	Yes	Yes	Yes	Yes	Yes	Yes	Yes	***8***
Lee et al. 2023 [37]	Yes	Yes	Yes	Yes	Yes	Yes	Yes	Yes	***8***
Logan et al. 2019 [38]	Yes	Yes	Yes	Yes	Yes	Yes	Yes	Yes	***8***
Meirizal et al. 2025 [39]	Yes	Yes	Yes	Yes	Yes	Yes	Yes	Yes	***8***
Miller et al. 2019 [40]	Yes	Yes	Yes	Yes	Yes	Yes	Yes	Yes	***8***
Mizutani et al. 2023 [41]	Yes	Yes	Yes	Yes	Yes	Yes	Yes	Yes	***8***
Mulier et al. 1993 [42]	Yes	Yes	Yes	Yes	Yes	Yes	Yes	Yes	***8***
Papanikolas et al. 2020 [7]	Yes	Yes	Yes	Yes	Yes	Yes	Yes	Yes	***8***
Rajeswari et al. 2009 [43]	Yes	Yes	Yes	Yes	Yes	Yes	Yes	Yes	***8***
Robbie et al. 2024 [44]	Yes	Yes	Yes	Yes	Yes	Yes	Yes	Yes	***8***
Rupp et al. 2018 [45]	Yes	Yes	Yes	Yes	Yes	Yes	Yes	Yes	***8***
Shimizu et al. 2015 [46]	Yes	Yes	Yes	Yes	Yes	Yes	Yes	Yes	***8***
Silveira et al. 2025 [47]	Yes	Yes	Yes	Yes	Yes	Yes	Yes	Yes	***8***
Siwach et al. 2009 [48]	Yes	Yes	Yes	Yes	Yes	Yes	Yes	Yes	***8***
Tan et al. 2021 [49]	Yes	Yes	Yes	Yes	Yes	Yes	Yes	Yes	***8***
Tsagozis et al. 2015 [50]	Yes	Yes	Yes	Yes	Yes	Yes	Yes	Yes	***8***
Vancabeke et al. 1999 [51]	Yes	Yes	Yes	Yes	Yes	Yes	No	Yes	***7***
Yonezu et al. 2025 [52]	Yes	Yes	Yes	Yes	Yes	Yes	Yes	Yes	***8***
Yoshikawa et al. 2019 [53]	Yes	Yes	Yes	Yes	Yes	Yes	Yes	Yes	8

Answer options include: “Yes”, “No”, “Unclear” or “Not applicable”; A1: Were patient demographic characteristics clearly described?; A2: Was the patient history clearly described and presented as a timeline?; A3: Was the current clinical condition clearly described?; A4: Were diagnostic tests or assessment methods and results clearly described?; A5: Was the intervention or treatment procedure clearly described?; A6: Was the post-intervention clinical condition clearly described?; A7: Were adverse events or unanticipated events identified and described?; A8: Does the case report provide takeaway lessons? ; Bold values under ‘Total Yes’ signify articles that satisfied all JBI quality measures.

**Table 9:** Quality assessment of the included case series using the Joanna Briggs Institute (JBI) critical appraisal checklist for case series

Reference (author, year)	B1	B2	B3	B4	B5	B6	B7	B8	B9	B10	Total yes (0–10)
Colosimo et al. 2020 [54]	Yes	Yes	Yes	Yes	Yes	Yes	Yes	Yes	No	No	8
Correa et al. 2008 [8]	Yes	Yes	Yes	No	No	Yes	Yes	Yes	Yes	No	7
Korambayil et al. 2021 [6]	Yes	Yes	Yes	Yes	Yes	Yes	Yes	Yes	No	Yes	9
Moura et al. 2017 [21]	Yes	Yes	Yes	Yes	Yes	Yes	Yes	Yes	No	Yes	9
Nuzzo et al. 1982 [55]	No	Yes	Yes	No	No	Yes	Yes	Yes	No	Yes	6
Schindler et al. 2023 [14]	Yes	Yes	Yes	Yes	Yes	Yes	Yes	Yes	No	No	8
Simman et al. 2022 [9]	No	Yes	Yes	No	No	Yes	Yes	Yes	No	Yes	6
Takahira et al. 2002 [56]	Yes	Yes	Yes	Yes	Yes	Yes	Yes	Yes	No	Yes	9
Wagstaff et al. 2019 [57]	Yes	Yes	Yes	Yes	Yes	Yes	Yes	Yes	No	Yes	9
Zalavras et al. 2009 [12]	Yes	Yes	Yes	Yes	Yes	Yes	Yes	Yes	No	Yes	9

Answer options include: “Yes”, “No”, “Unclear” or “Not applicable”; B1: Were there clear criteria for inclusion in the case series?; B2: Was the condition measured in a standard, reliable way for all participants?; B3: Were valid methods used for identification of the condition for all participants?; B4: Did the case series have consecutive inclusion?; B5: Did the case series have complete inclusion?; B6: Was there clear reporting of demographics?; B7: Was there clear reporting of clinical information?; B8: Were outcomes or follow-up results clearly reported?; B9: Was there clear reporting of presenting sites/clinics?; B10: Was statistical analysis appropriate?

**Table 10:** Modified Newcastle-Ottawa Scale appraisal of retrospective cohort/database studies

Study	Selection (0–4)	Comparability (0–2)	Outcome (0–3)	Total (0–9)	Key limitation
Endean et al. 1991 [10]	3	0	2	5	Historical retrospective cohort; deaths not consistently linked to specific infectious subtype; limited confounding control
Schwartz et al. 2020 [3]	4	1	2	7	Administrative database; small-cell suppression; limited microbiology, acuity, and functional outcome data
Unruh et al. 1990 [13]	3	0	1	4	Historical mixed cohort; parent-cohort demographics; limited follow-up detail and confounder adjustment

## Discussion

This systematic review synthesizes the available literature on HD and EH performed for infectious indications, emphasizing the denominator and reporting limitations that constrain interpretation. The analytic cohort was dominated by PJI, but the evidence base combined individual case reports, small case series, historical cohorts, and one large administrative database study. After separating patient-level from procedure-level denominators, the all-study upper-bound early mortality was 12.8% to < 16.6%, whereas mortality rose to 26.2% when the largest registry cohort was excluded. Functional outcome data were available for only 67 survivors, and nearly two-thirds of that subset remained wheelchair-dependent or bedbound. These findings reinforce the role of HD and EH as last-resort source-control procedures while underscoring that published outcomes cannot be generalized without attention to study type, mortality window, and follow-up completeness.

### Mortality rates

Our all-study mortality estimate is heavily influenced by the Schwartz et al. cohort [3], which contributed 148 of 289 patients but reported only a suppressed in-hospital death count of < 11. When that cohort was retained, early mortality could only be expressed as a range or upper bound (37 to < 48/289, 12.8% to < 16.6%). When it was excluded, early mortality was substantially higher (37/141, 26.2%).

Differences across studies likely reflect both patient acuity and era-specific care. Endean et al. reported 14% mortality for infection alone and 33% with concurrent ischemia [10], while Unruh et al. reported 52% mortality in the setting of preoperative infection [13]. More recent series suggest that multidisciplinary care, aggressive source control, and staged surgical strategies can improve survivorship. Zalavras et al. reported 6.7% mortality in severe lower extremity infections [12], and Schwartz et al. reported an in-hospital mortality rate below 7.4% for PJI-related HD [3]. Smaller case series describing staged damage-control for NSTI and chronic osteomyelitis similarly report favorable survival to discharge [8, 54].

Despite these advances, mortality remains high when patients present with fulminant sepsis, ischemia, or irreversible physiological collapse. Recent series by Korambayil et al. and Schindler et al. reported mortality rates of 40% and 57%, respectively, among severe infectious cases [6, 14]. Importantly, the available literature may underestimate true mortality because many reports capture only in-hospital outcomes, several case reports have follow-up of 12 h to 44 days, and late recurrent sepsis or failure of source control may not be captured. The mortality estimates in this review should therefore be used for counseling as early or index-episode estimates rather than as definitive long-term survival probabilities.

### Long-term outcomes

Survivors of HD and EH face profound functional challenges, but the published functional evidence is sparse. A prior prosthetic scoping review emphasized the limited evidence base for HD and hemipelvectomy prostheses [16]. In the present infection-specific review, functional status was reported for only 67 survivors, with follow-up ranging from 21 days to 10 years. Consequently, the observed finding that 62.7% remained wheelchair-dependent or bedbound should be interpreted as a descriptive subset estimate rather than a generalizable cohort outcome. Early follow-up may understate eventual rehabilitation gains, while case reports may preferentially publish unusual successes or catastrophic failures.

To mitigate the physiological burden, surgical and rehabilitative innovations aim to optimize the postoperative limb for sitting balance, transfers, socket tolerance, and selective prosthetic use. Surgical techniques such as creation of a functional above-knee stump using modular femoral replacement [26] or pedicled fillet flaps [57] with biodegradable temporizing matrices [57] have been described to improve soft-tissue durability and prosthetic fit. Rehabilitation using body-weight-support systems can reduce the metabolic cost of gait training [40]. Nevertheless, across the limited functional subset summarized in Table 7, independent community ambulation remained uncommon, and most reported survivors required a wheelchair, bed-level care, assistive devices, or substantial rehabilitation support.

### Infectious indications

#### Prosthetic joint infection

PJI represents the most frequent and rapidly growing indication for HD, with national registry data documenting a 366% increase in procedural volume between 2002 and 2016 [3]. Independent risk factors for requiring HD over revision arthroplasty include peripheral vascular disease (OR 2.59), diabetes with chronic complications (OR 1.91), and lacking private insurance under age 65 [3]. The interplay of vascular insufficiency and infection is particularly deleterious; patients with severe peripheral arterial disease who fail revascularization are often left with HD as the only viable option to prevent ascending necrosis and sepsis [37].

The clinical trajectory and mortality of HD for PJI are heavily influenced by surgical urgency. While elective procedures carry a reported mortality of approximately 4%, this rate rises to 33% when performed emergently [21]. The delays in radical intervention that often stem from a reluctance to perform disarticulation in favor of repeated, less aggressive limb-salvage attempts can precipitate uncontrolled pelvic sepsis and shock [24]. Microbiologically, while *Staphylococcus* and *Pseudomonas* species are most frequently implicated [21, 35], atypical isolates such as *Clostridium septicum* mandate rigorous screening for occult gastrointestinal malignancy [5].

#### Necrotizing fasciitis and necrotizing soft tissue infections

While the literature frequently uses necrotizing fasciitis and necrotizing soft tissue infections interchangeably, both describe a spectrum of rapidly progressive, toxin-mediated emergencies necessitating immediate decision-making. These infections carry exceptionally high mortality rates, ranging from 40% [6] to over 63% when involving the deep compartments of the lower limb [7]. Survival is strictly contingent upon aggressive source control, but diagnosis is often obscured by atypical presentations. In patients with spinal cord injuries, for instance, sensory deficits can mask the hallmark symptom of pain out of proportion to exam [24]. Furthermore, a distinct and lethal “descending” pattern occurs when intra-abdominal pathologies such as perforated appendicitis, diverticulitis, or colorectal malignancies breach the retroperitoneum and track through the psoas or femoral canal into the thigh without an initial cutaneous breach [7, 44, 45].

The microbiological spectrum driving these amputations ranges from monomicrobial Group A *Streptococcus* [24, 30] to synergistic polymicrobial anaerobes [6]. Comorbidities such as immunosuppression, diabetes, and intravenous drug use act as critical accelerators for tissue destruction [6, 34, 43]. Studies in this review highlight a stark contrast in outcomes based on surgical strategy: delays in achieving source control uniformly lead to multiorgan failure and death [52], whereas a two-stage “damage control” approach (rapid guillotine amputation followed by delayed closure) yielded up to 100% survival to discharge in a modern NSTI cohort [54].

#### Osteomyelitis

In contrast to the acute nature of NSTIs, HD and EH serve as definitive “rescue procedures” for chronic, recalcitrant osteomyelitis of the proximal femur and pelvis. These cases represent the culmination of years of failed conservative management, including repeated debridement and prolonged hospitalization, most notably in paraplegic patients with non-healing pressure ulcers [8, 9, 51]. In this setting, the surgical goal extends beyond survival to the restoration of functional independence; removing the septic focus significantly reduces systemic inflammatory burden and facilitates a return to wheelchair mobility [8, 26]. The bacteriology is typically polymicrobial, involving multidrug-resistant organisms such as *Proteus mirabilis*, *Pseudomonas aeruginosa*, and *Staphylococcus aureus*, which require broad-spectrum coverage and robust soft-tissue flap reconstruction [9, 39, 51]. A vital diagnostic caveat in this cohort is the risk of Marjolin’s ulcer (squamous cell carcinoma) arising from chronic wounds. This malignant transformation can masquerade as refractory osteomyelitis or even mimic an acute NSTI with gas formation, underscoring the necessity of aggressive biopsy to prevent delays in oncologic clearance [33, 36].

#### Gas gangrene

Gas gangrene remains one of the most rapidly lethal indications for radical amputation. It typically presents in two forms: trauma-associated *Clostridium perfringens* [38] and atraumatic, hematogenously spread *Clostridium septicum*. The isolation of *C. septicum* is highly associated with occult colorectal carcinoma or neutropenic enterocolitis, making early gastrointestinal screening essential [5, 28, 34]. Mortality in this subgroup is exceedingly high, reported between 32% and 100% depending on the speed of intervention [11, 28]. Crucially, the presence of gas in the soft tissues is not exclusive to clostridial species; non-clostridial gas gangrene caused by facultative anaerobes (*Klebsiella pneumoniae*, *Escherichia coli*) is frequently documented in diabetic patients with severe microangiopathy [27, 56]. The presence of myonecrosis and crepitus, regardless of the specific organism, necessitates immediate surgical intervention as antibiotic therapy alone is universally fatal in the face of rapid, toxin-mediated tissue liquefaction [11, 56].

#### Atypical and rare indications

Beyond common bacterial pathogens, a spectrum of atypical organisms and pathologies can necessitate HD in specific geographic or traumatic contexts. Melioidosis (*Burkholderia pseudomallei*), endemic to tropical regions, can progress to refractory osteomyelitis and sepsis despite appropriate antibiotics, requiring amputation for survival [49]. Similarly, angioinvasive fungal infections such as mucormycosis (*Rhizopus* species) can contaminate complex blast injuries, leading to accelerated tissue necrosis that outpaces serial debridement and mandates urgent disarticulation [23]. Other rare presentations include *Arcanobacterium haemolyticum* NF in intravenous drug users [29] and rapidly fatal *Aeromonas hydrophila* myonecrosis in immunocompromised hosts [52]. Skeletal hydatidosis (*Echinococcus granulosus*) can mimic locally aggressive pelvic tumors, erode bone, and require en-bloc resection to prevent anaphylaxis from cyst rupture [31, 48, 50]. Lastly, benign lesions such as chronic expanding hematomas can become secondarily infected, transforming into massive, life-threatening abscesses that demand radical amputation for source control [41].

### Limitations

This review is limited by the predominance of low-level evidence, comprising primarily case reports, small case series, historical retrospective cohorts, and one large administrative database study. Case reports were included because the condition is rare, but their inclusion introduces publication bias and limits comparability with registry data; sensitivity analyses were therefore added to separate case reports, aggregate studies, and the largest registry cohort. Denominator instability is a major limitation: the analytic cohort includes 289 patients, procedure summaries include 291 procedures because two patients underwent revision EH after HD, and sex could be summarized only for 260 analytic-cohort patients because two studies reported sex only for their parent cohorts. Mortality definitions were inconsistent across studies, with in-hospital, immediate postoperative, 30-day, and longer follow-up windows variably reported. Several reports had very brief follow-up (including 12 h, 21 days, and 44 days), creating a substantial risk of underestimating late mortality, recurrent infection, and delayed complications. Functional outcomes were reported for only 67 survivors and cannot be generalized to the entire cohort. Microbiology was incompletely reported and frequently polymicrobial; aggregate studies often provided isolate-level rather than patient-level data, precluding valid pooled microbiology percentages. The English-language restriction introduces language bias, and deviations from the registered protocol occurred because Scopus and formal forward-citation screening were not completed and complications/health-related quality-of-life outcomes were too sparsely reported for meaningful synthesis. Finally, JBI and modified NOS appraisals identify reporting and methodological limitations but cannot remove confounding effects by illness severity, selection bias, or era effects. No causal inferences should be drawn from the descriptive data presented in this review.

## Conclusion

HD and EH remain rare but necessary salvage procedures for severe musculoskeletal infections that are refractory to limb-sparing interventions. Mortality is substantial, but the available literature does not support a single precise pooled estimate because study designs, mortality windows, and follow-up duration vary widely. Patient-level sensitivity analyses suggest early mortality below 16.6% when all studies are retained and approximately 26% when the largest registry cohort is excluded. Functional recovery is sparsely reported however, among documented survivors, long-term wheelchair dependence or bedbound status is common. Multidisciplinary source control, critical care, antimicrobial management, rehabilitation planning, and realistic patient-family counseling remain essential when considering these high-stakes operations.

## Supplementary Information

Below is the link to the electronic supplementary material.

Supplementary file 1

## Data Availability

The data supporting the findings of this study were extracted from previously published articles, which are cited in the reference list. Extracted study-level data and analytic code are available from the corresponding author upon reasonable request.
