# Supplementary material for: Hip disarticulation and external hemipelvectomy for infectious indications: a systematic review
Source: Eur J Orthop Surg Traumatol. 2026 Jun 15;36(1):241. doi: 10.1007/s00590-026-04827-6 (PMC13269480; doi:10.1007/s00590-026-04827-6)
Supplement: Supplementary file 1 [file 590_2026_4827_MOESM1_ESM.docx]

**Supplementary Appendix 1**

**Search:** up to 3 January 2026

**PubMed =** 421

(("hip disarticulation"[MeSH Terms] OR "hip disarticulation"[Title/Abstract] OR "hindquarter amputation"[Title/Abstract] OR "hemipelvectomy"[MeSH Terms] OR "hemipelvectomy"[Title/Abstract])

AND

("infection"[MeSH Terms] OR "infection"[Title/Abstract] OR "necrotizing fasciitis"[MeSH Terms] OR "necrotizing fasciitis"[Title/Abstract] OR "gas gangrene"[MeSH Terms] OR "gas gangrene"[Title/Abstract] OR "clostridium infections"[MeSH Terms] OR "clostridial myonecrosis"[Title/Abstract] OR "periprosthetic joint infection"[Title/Abstract] OR "osteomyelitis"[MeSH Terms] OR "osteomyelitis"[Title/Abstract] OR "sepsis"[MeSH Terms] OR "sepsis"[Title/Abstract]))

**Embase =** 527

('hip disarticulation'/exp OR 'hip disarticulation' OR 'hemipelvectomy'/exp OR hemipelvectomy OR 'hindquarter amputation')

AND

('infection'/exp OR infection OR 'infectious disease'/exp OR 'infectious' OR 'necrotizing fasciitis'/exp OR 'necrotizing fasciitis' OR 'necrotizing soft tissue infection' OR 'gas gangrene'/exp OR 'gas gangrene' OR 'clostridial myonecrosis' OR 'periprosthetic joint infection' OR 'osteomyelitis'/exp OR osteomyelitis OR 'sepsis'/exp OR sepsis)

**Web of Science =** 237

(("hip disarticulation" OR "hemipelvectomy" OR "hindquarter amputation")

AND

("infection" OR "infectious" OR "necrotizing fasciitis" OR "necrotizing soft tissue infection" OR "gas gangrene" OR "clostridial myonecrosis" OR "periprosthetic joint infection" OR "osteomyelitis" OR "sepsis"))

**Cochrane Central Register of Controlled Trials** = 3

("hip disarticulation" OR "hemipelvectomy" OR "hindquarter amputation")

AND

("infection" OR "infectious" OR "necrotizing fasciitis" OR "necrotizing soft tissue infection" OR "gas gangrene" OR "clostridial myonecrosis" OR "periprosthetic joint infection" OR "osteomyelitis" OR "sepsis")

**Total= 1188**
